# Influenza-attributable deaths in south-eastern France (1999 to 2010): mortality predictions were undependable

**DOI:** 10.1186/s12889-015-1887-y

**Published:** 2015-06-07

**Authors:** Simon-Djamel Thiberville, Jean Gaudart, Didier Raoult, Remi N. Charrel

**Affiliations:** Aix Marseille Université, IRD French Institute of Research for Development, EHESP French School of Public Health, EPV UMR_D 190 “Emergence des Pathologies Virales”, 13385 Marseille, France; IHU Méditerranée Infection, APHM Public Hospitals of Marseille 13385, Marseille, France; Aix-Marseille Univ, UMR912, SESSTIM (AMU, IRD, INSERM), Marseille, France

**Keywords:** Influenza mortality, Pandemic influenza A(H1N1)2009, Influenza attributable deaths, Influenza in France

## Abstract

**Background:**

Following the 2009 influenza pandemic, several studies showed that the mortality pattern associated with the A(H1N1)2009 virus primarily affected children and young adults. In this study, we aimed to estimate the influenza-attributable deaths during the periods from 1999 to 2010, in the Provence-Alpes-Côte-d’Azur (PACA) region of south-eastern France in order to corroborate the hypothesis that (i) influenza-attributable deaths caused by A(H1N1)2009 strain were much lower than initially expected.

**Methods:**

In order to compare our results with published data, we used the same statistical model of an Austrian team, using a Poisson model adjusted on co-circulating respiratory syncytial virus to explain the weekly mortality.

**Results:**

We assessed that 5.7 % of the respiratory deaths were attributable to influenza virus during the 2009–2010 pandemic season. This mortality was lower than that observed during the ten preceding epidemic periods (13.8 % on average). Age group- based analysis revealed that during the pandemic period, the groups under 65 had a systematically higher excess of respiratory mortality while the age group over 65 had a much lower mortality than during the seasonal epidemic seasons. Similarly, among the less specific outcome (non violent and cardiovascular mortality) the age groups over 45 had higher excess of mortality during the seasonal epidemics than during the pandemic period.

**Conclusions:**

Since most of the influenza mortality is commonly observed in the elderly group (>65 year-old), the moderate elderly mortality during the 2009 pandemic period has impacted the total mortality, and has resulted in a reduced total mortality despite an increased mortality in the young age group. Despite using identical parameters and the same approach as in a previously published study using an Austrian population sample, we observed a lower excess respiratory mortality in the south-eastern France than in Vienna. Thus, the pandemic virus caused less death than the epidemic viruses that circulated during the preceding years. In contrast with catastrophic predictions made in the early phase of the pandemic, human lives were saved during the circulation period of A(H1N1)2009 virus, resulting in a lower overall mortality.

## Background

In April 2009, a novel influenza A(H1N1)2009 virus emerged in Mexico and spread rapidly worldwide leading the World Health Organization (WHO) to declare the first influenza pandemic of the twenty-first century [1]. Soon after, dreadful predictions were made and amplified by maintained confusion between H5N1 and H1N1 viruses and vaccine safety issues in scientific and public media [2–4]. The first studies on the mortality of the new pandemic influenza virus showed that children and young adults were primarily affected [5,6]. In contrast, elderly were protected due to previous infection with an antigenically-related influenza virus during youth [7]. Assessing the number of deaths attributable to influenza epidemics is difficult because most of deaths occur after the acute phase of viral infection from bacterial super-infection or from complications of chronic illnesses. Accordingly, in both cases the primary viral infection is no longer directly detectable. A commonly accepted approach consists of using statistical time series seasonal regression models of different causes of death recorded by national system [8]. In an Austrian study, mortality attributable to A(H1N1)2009 influenza virus adjusted on co-circulating respiratory syncytial virus (RSV) in the area of Vienna, Austria was lower during the pandemic than during the preceding epidemic periods (1999/2000–2008/2009). Attributable mortality increased significantly for the age groups below 34 years old and decreased significantly for those above 55 during the pandemic compared to the epidemics periods [9].

Using the same approach, we estimated the seasonal influenza-attributable deaths from 1999 to 2009, and the pandemic influenza-attributable deaths in 2010, in the Provence-Alpes-Côte-d’Azur (PACA) region of south-eastern France in order to corroborate the hypothesis that (i) influenza-attributable deaths caused by A(H1N1)2009 strain were much lower than initially expected, (ii) and that A(H1N1)2009 virus primarily affected young people [10].

## Methods

### Mortality data and study population

We used the weekly reported deaths of the PACA region south-eastern France, from week 52, 1999 through week 51, 2010 collected by INSERM CépiDc [11]. The file contained gender, age at death grouped in 5 year ranges, week of death, and cause of death using the four digit International Classification of Diseases (ICD) code (10th revision).

Population of PACA was 4.5 millions in 1999 and 4.9 millions in 2010 according to the French National Institute of Statistics and Economic Studies [7].

### Surveillance of Influenza virus infections

Influenza activity is mainly monitored in France through the sentinel practitioner network [12] that records the weekly number of influenza-like illness (ILI) syndrome, defined as sudden abrupt onset fever above 39 °C AND respiratory signs AND myalgia [13].

The onset of the influenza epidemic period was defined as the first week with ILI count (per 100,000 inhabitants) above the threshold value.

The end of the influenza epidemic period was defined as the last week with ILI count (per 100,000 inhabitants) below the threshold value.

The threshold was defined from non-epidemic baseline of ILI count, forecasted by the sentinel networks [13].

The most prevalent type and subtype of influenza viruses that circulated each year were defined by the national reference centre for influenza [5].

### Respiratory syncytial virus (RSV) surveillance

Nasopharyngeal aspirates (NPA) or nasal swabs (NS) were obtained from patients admitted to the Public Hospital of Marseille, France, who were clinically suspect for RSV. Direct detection of RSV was performed by real-time RT-PCR as described previously [14].

### Statistical analysis

In order to compare our results with published data, we used the same statistical model used by Redlsperger-Fritz [9]. General linear models (glm) were used to estimate the expected number of deaths according to a Poisson distribution model. The population log was used as an offset. Cofactors were the total number of RSV, the epidemic weeks (dummy variable), and the sinusoidal transformation of time. The attributable number of deaths AD_j_ for epidemic weeks j were estimated as following:

$$ \mathrm{A}{\mathrm{D}}_{\mathrm{j}}={\mathrm{D}}_{\mathrm{j}}\left(1- \exp \left\{-{\uptheta}_{\mathrm{j}}\right\}\right) $$

Where, for the epidemic week j, D was the number of deaths and θ the associated parameter estimated by the glm. The dependent variables were the weekly deaths from all non-violent causes (all codes except ICD9: 800–999, ICD10: S/T), from respiratory diseases (ICD9: 460–519, ICD10: J), and from cardiovascular diseases (ICD9: 390–459, ICD10: I). To assess the excess mortality by age group, we used 4 age groups (0–24, 25–44, 45–64 and > 65 year-old).

All statistics were provided by using the R software (version R3.1.0; http://www.r-project.org/).

## Results

During the studied period, 470,957 non violent deaths were registered, including 31,688 respiratory- and 142,352 cardiovascular- related deaths.

A total of 1,862,815 ILI were recorded during the eleven influenza waves (ten epidemic seasons and one pandemic season), and 443,376 ILI were recorded during the non-epidemic weeks. On average, 169,346 cases of ILI were recorded per epidemic/pandemic season (range 81,425–266,313). The mean number of epidemic/pandemic weeks per year was ten, ranging from 7 (2006/2007) to 19 (2009/2010).

According to the National Reference Centre of Influenza, 6/10 epidemic seasons witnessed circulation of A/H3N2 virus subtype, whereas A/H1N1 virus subtype and B virus type co-circulated during the 4 remaining epidemic seasons. During the pandemic period virus circulation was due almost exclusively to A(H1N1)2009 influenza.

A total of 24,329 samples were tested for RSV in the virology laboratory, of which 3,676 (15.1 %) were positive for RSV RNA. The mean yearly number of RSV-positive samples was 333 (range 81–565).

We estimated the excess of mortality (non violent, respiratory, and cardiovascular) during the influenza epidemic periods in the population of the studied region, and during each epidemic year (from 1999–2000 to 2009–2010) (Table 1).

**Table 1:** Excess mortality (per 100,000 inhabitants) attributable to influenza virus per season from 1999/2000 to 2009/2010 in the Provence-Alpes-Côte-d’Azur (PACA) region, France

Epidemic season of influenza	Non violent mortality N [CI] (per 100,000)	Respiratory mortality N [CI] (per 100,000)	Cardiovascular mortality N [CI] (per 100,000)
1999/2000	198 [106;370] (4.36)	116 [81;171] (2.55)	57 [−4;150] (1.26)
2000/2001	184 [77;365] (4.01)	77 [49;117] (1.68)	54 [−16;153] (1.18)
2001/2002	190 [71;388] (4.10)	93 [57;145] (2.01)	54 [−23;165] (1.17)
2002/2003	291 [83;633] (6.22)	109 [54;182] (2.33)	50 [−81;248] (1.07)
2003/2004	195 [88;370] (4.13)	94 [61;139] (1.99)	52 [−13;146] (1.10)
2004/2005	275 [109;563] (5.77)	139 [82;221] (2.91)	58 [−44;215] (1.22)
2005/2006	171 [67;344] (3.55)	65 [40;99] (1.35)	48 [−17;139] (1.00)
2006/2007	147 [54;310] (3.02)	79 [52;119] (1.62)	41 [−16;124] (0.84)
2007/2008	173 [63;355] (3.54)	73 [43;116] (1.50)	48 [−19;142] (0.98)
2008/2009	317 [141;624] (6.48)	107 [54;180] (2.19)	56 [−45;219] (1.15)
2009/2010	274 [−324;996] (5.59)	55 [−115;206] (1.12)	−113 [−460;266] (−2.31)

During the ten influenza epidemic seasons, the excess respiratory mortality ranged from 1.35 to 2.91 per 100,000 inhabitants corresponding to an average 13.8 % of the respiratory deaths (12.7–15.2 %). An excess of respiratory mortality could be attributable to the influenza subtype with an average mortality of 2.21 and 1.71 per 100,000 for the A/H3N2 subtype seasons on the one hand and A/H1N1-B subtypes seasons on the other hand, respectively. In contrast, during the 2009 pandemic season, a non-significant excess of respiratory deaths (55; 95 % CI[−115;206]) was attributable to the A(H1N1)2009 pandemic period corresponding to an excess respiratory mortality of 1.12 per 100,000 (5.7 % of the respiratory deaths).

Among the less specific outcome, the excess mortality during the epidemic seasons ranged from 3.02 to 6.48 and from 0.84 to 1.26 per 100,000 for the non violent and cardiovascular mortality respectively. During the pandemic period, the excess of non violent mortality was similar to the excess of non violent mortality during epidemic periods (5.59 per 100,000), but this excess was not significant. Furthermore, a non significant negative excess of mortality (−2.31 per 100,000) was estimated for cardiovascular mortality.

**Fig. 1:**
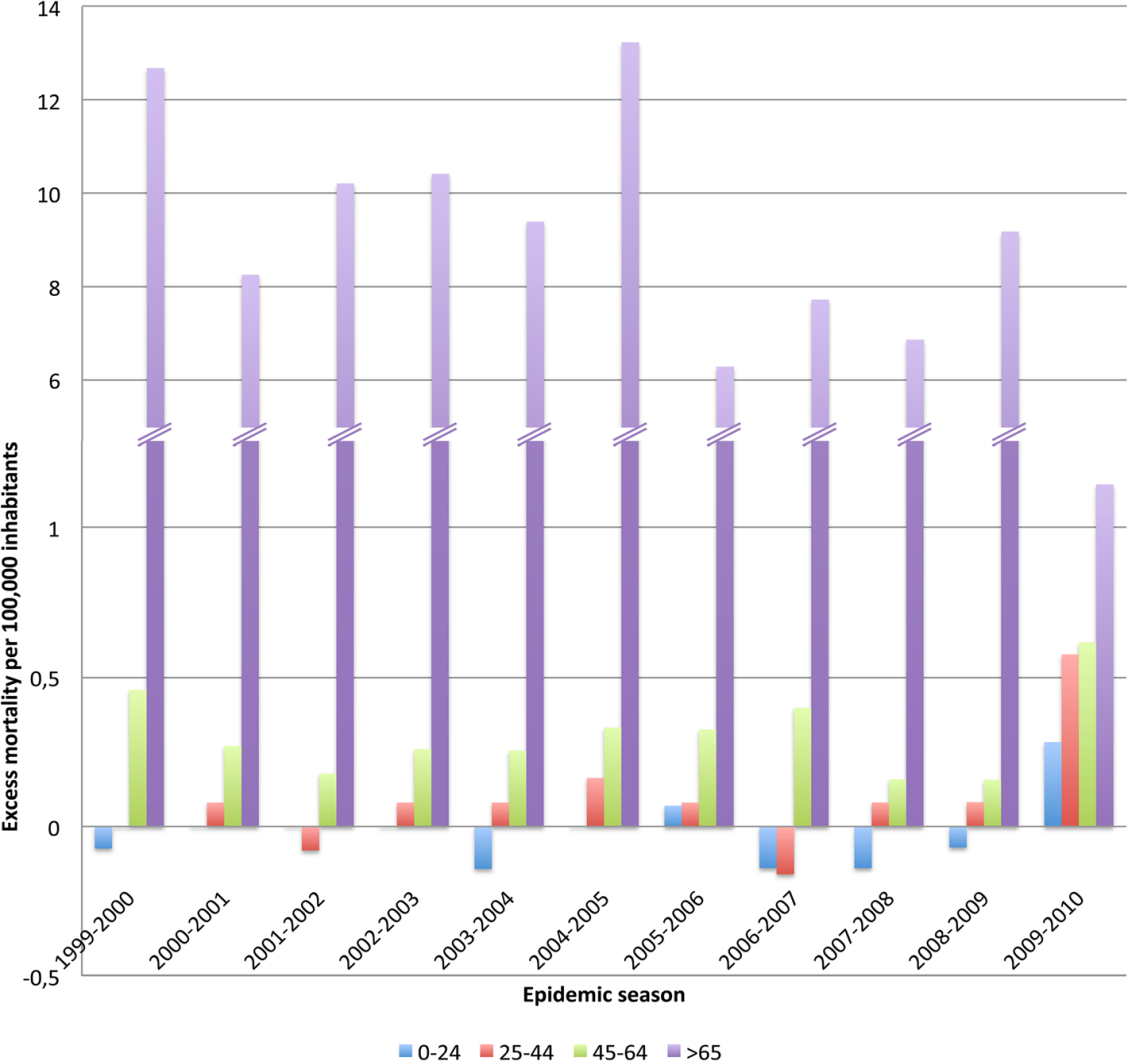
Excess influenza respiratory mortality (per 100,000 inhabitants) by group of age (0–24 in blue, 25–44 in red, 45–64 in green and over 65 years old in purple) estimated during the 2009 pandemic and the ten preceding epidemics in the Provence-Alpes-Côte-d’Azur (PACA) region, France. The method use to assess the mortality attributable to influenza virus was previously described [9]

Age group- based analysis (Fig. 1) revealed an excess respiratory mortality during seasonal epidemic seasons with the following ranges: −0.14 - 0.07, −0.16 - 0.16, 0.16 - 0.46, and 6.27 - 13.23 per 100,000 observed in the respective age groups 0–24, 25–44, 45–64 and > 65 year-old. In contrast during the pandemic period, the groups under 65 had a systematically higher excess of respiratory mortality (0.28, 0.58 and 0.62 for the age groups 0–24, 25–44 and 45–64 respectively) while the age group over 65 had a much lower mortality (1.14 per 100,000).

For non violent and cardiovascular mortality the age groups over 45 had higher excess of mortality during the seasonal epidemics (45–64 years: ranging from −1.46 to 0.18 for non violent mortality and from 0.58 to 0.81 for cardiovascular mortality; over 65: ranging from 15.10 to 26.14 for non violent mortality and from 2.40 to 5.86 for cardiovascular mortality) than during the pandemic period (45–64 years: −3.77 for non violent mortality and −0.69 for cardiovascular mortality; over 65: 2.91 for non violent mortality and −19.03 for cardiovascular mortality).

## Discussion

We assessed that respiratory mortality attributable to influenza was lower during the pandemic period than during the epidemic seasons. Since most of the influenza mortality is commonly observed in the elderly group (>65 year-old), the moderate elderly mortality during the 2009 pandemic period has impacted the total mortality, and has resulted in a reduced total mortality despite an increased mortality in the young age group.

Similarly, in Austria, Redlsperger-Fritz *et al.* reported an excess mortality in the young age groups (<34 year-old) and a reduced mortality in age groups > 55 year-old during the 2009 pandemic period, compared with the epidemic seasons [9]. In France, Lemaitre *et al.* showed that the mortality burden of the 2009 pandemic was particularly mild except in the age groups 5 to 44 years old [5].

The reasons for this low mortality among elderly may relate to the existence of prior immunity of individuals born before 1957 after infection with an A(H1N1)2009 antigenically related virus [15,16]. The specific age-related 2009 pandemic mortality pattern which was also noticed in previous influenza pandemics (1957, 1968) is currently considered as a signature of the influenza pandemics [5].

Since cardio-vascular diseases are reduced by the influenza vaccination in elderly patients [17], we considered it interesting to analyse the excess cardiovascular mortality attributable to influenza. Interestingly, as showed for the respiratory mortality, the excess cardiovascular mortality during the epidemic seasons was higher than during the pandemic period during which a non significant negative excess of mortality was observed. Pre-existing exposure to closely related viruses prior to 1957 may have provided cross-reactive immunity during the A(H1N1)2009 outbreak in the same way influenza vaccination may reduce the incidence of influenza-related complications [17].

In our study, the mortality was dependent on the predominant influenza subtype circulating: A/H3N2 was associated with higher mortality than A/H1N1 and B influenza virus. The same trend was identified in the Austrian study [9]. A higher mortality was also observed in all age groups with the A(H3N2)1968 pandemic compared to the A(H1N1)2009 pandemic [5].

Although most countries reported a lower impact on the overall mortality during the 2009 pandemic compared to the previous seasonal mortality, the number of excess deaths attributable to influenza was highly variable in the literature [5,6]. Despite using identical parameters such as respiratory mortality and the same age classes, great variations were observed in excess mortality from 4.1 per 100,000 in France to 10.5 per 100,000 in Mexico during the 2009 pandemic [5,6]. Whether some of these differences between countries and studies may reflect differences in prior immunity, health care, climate variations, co-circulation of other viruses or was due to the use of different statistical models remains to be clarified.

Here, we used an approach identical to that used by Redlsperger-Fritz *et al.* in Austria, incorporating RSV circulation as a covariate [9]. However, in the elderly age group we observed a lower excess respiratory mortality in south-eastern France than in Vienna (1.12 vs 10.8 per 100,000 inhabitants during the A(H1N1)2009 pandemic [9]. Moreover, as Lemaitre *et al.* previously reported, we also found a negative attributable mortality during the pandemic period for the less specific outcome in the analysis by age group [5]. This may be due to the particularly mild mortality impact of the pandemic 2009 in France, which, with regards to non specific mortality, was overflowed by the mortality attributable to other cause [11].

The mortality attributable to the influenza epidemics has been the subject of numerous front line articles but none could forecast that the most recent influenza pandemic had been associated with a very low mortality impact [18] whereas during the same period the influenza vaccination coverage was historically low in the general population in France [19]. Immune protection due to past exposure to antigenetically related viruses is important when estimating/modeling the disease impact of a new viral threat. The A(H1N1)2009 pandemic could have had serious consequences, had the age group of the elderly not been partially immune to the virus. Using an effective vaccine in the children on the other hand, could have major impact on preventing spread of pandemic viruses – including to other risk groups, such as the elderly, as previously demonstrated [20].

## Conclusion

Compared to previous seasonal influenza epidemics, the 2009 influenza pandemic has resulted in a reduction of the total mortality, due to a reduced death rate in the elderly population; the introduction of the novel A(H1N1)2009 pandemic virus has saved lives.
